# Interfacial Engineering of Leaf-like Bimetallic MOF-Based Co@NC Nanoarrays Coupled with Ultrathin CoFe-LDH Nanosheets for Rechargeable and Flexible Zn-Air Batteries

**DOI:** 10.3390/polym15030734

**Published:** 2023-01-31

**Authors:** Jinliang Ma, Longlong Liu, Zihao Chen, Minghui Wang, Han Wu, Hongmiao Wang, Ding Yuan, Xin Ning

**Affiliations:** Industrial Research Institute of Nonwovens & Technical Textiles, Shandong Center for Engineered Nonwovens, College of Textiles & Clothing, Qingdao University, Qingdao 266071, China

**Keywords:** carbon fiber, bifunctional electrocatalysts, bimetallic MOFs, self-supporting electrodes, Zn-air battery

## Abstract

Exploring high-efficiency, low-cost, and long-life bifunctional self-supporting electrocatalysts is of great significance for the practical application of advanced rechargeable Zn-air batteries (ZABs), especially flexible solid-state ZABs. Herein, ultrathin CoFe-layered double hydroxide (CoFe-LDH) nanosheets are strongly coupled on the surface of leaf-like bimetallic metal-organic frameworks (MOFs)-derived hybrid carbon (Co@NC) nanoflake nanoarrays supported by carbon cloth (CC) via a facile and scalable method for rechargeable and flexible ZABs. This interfacial engineering for CoFe-LDHs on Co@NC improves the electronic conductivity of CoFe-LDH nanosheets as well as achieves the balance of oxygen evolution reduction (OER) and oxygen reduction reaction (ORR) activity. The unique three-dimensional (3D) open interconnected hierarchical structure facilitates the transport of substances during the electrochemical process while ensuring adequate exposure of OER/ORR active centers. When applied as an additive-free air cathode in rechargeable liquid ZABs, CC/Co@NC/CoFe-LDH-700 demonstrates high open-circuit potential of 1.47 V, maximum power density of 129.3 mW cm^−2^, and satisfactory specific capacity of 710.7 mAh g^−1^_Zn_. Further, the flexible all-solid-state ZAB assembled by CC/Co@NC/CoFe-LDH-700 displays gratifying mechanical flexibility and stable cycling performance over 40 h. More significantly, the series-connected flexible ZAB is further verified as a chain power supply for LED strips and performs well throughout the bending process, showing great application prospects in portable and wearable electronics. This work sheds new light on the design of high-performance self-supporting non-precious metal bifunctional electrocatalysts for OER/ORR and air cathodes for rechargeable ZABs.

## 1. Introduction

With the rapid growth of energy demand and the ever-worsening of environmental issues, it is extremely important to explore new energy storage and conversion devices that are economical, efficient, and eco-friendly [1,2,3,4]. Concurrently, electronics are developing towards portability and flexibility in recent years, posing a major challenge to flexible wearable energy storage devices [5,6,7]. As one of the next-generation alternative energy technologies, rechargeable Zn-air batteries (ZABs) have broad application prospects in future electric vehicles, portable electronic products, flexible wearable electronic devices, and off-grid power supplies due to their large theoretical energy density, high safety, abundant resources, and environmental amity [6,8,9]. Nevertheless, the actual energy efficiency and cycling performance of ZABs are still far from satisfactory at this time, which is attributed to the poor reactivity of oxygen evolution reduction (OER) and oxygen reduction reaction (ORR) on the air cathode during the charge-discharge process [10,11,12,13,14]. These two electrochemical reactions are multi-electron transfer processes with complex reaction paths, resulting in high overpotential and sluggish kinetics [15,16]. Traditional precious metal-based materials, such as platinum (Pt), iridium (Ir), and ruthenium (Ru), have been confirmed as highly efficient electrocatalysts for ORR or OER, but their large-scale application in ZABs is greatly hindered by the disadvantages of scarcity, expensiveness, single functionality, and poor durability [10,17]. Furthermore, in order to coat these powdery electrocatalysts on the surface of the air cathode, non-conductive polymer binders are often used to enhance the bonding strength [18,19]. However, it is easy to cause problems such as reduction of electrical conductivity, blockage of pores, or coverage of active sites [18,20]. Therefore, it is of economic value and scientific interest to exploit self-supporting bifunctional electrocatalysts (OER/ORR) with low cost, high efficiency, and stability.

Over the past few decades, a series of earth-abundant transition metals and carbon-based materials comprising unique properties and structures have been developed to substitute for noble metal-based catalysts [21,22]. Among them, the transition metal-nitrogen-carbon (TM-N-C, TM = Co, Fe, Ni, Mn, etc.) systems are considered to be the most promising catalysts because of their high catalytic activity, cost-efficient and good durability in alkaline environments [23,24,25]. In particular, TM-N-C electrocatalysts derived from metal-organic frameworks (MOFs), which possess large surface area, exceptional porosity, uniformly dispersed metal centers, and abundant nitrogen sources, have attracted enormous attention [24,26]. So far, several pioneering works have demonstrated that MOF-derived carbon-based materials, for example, zeolitic imidazolate frameworks (ZIFs) derivatives, can serve as outstanding ORR catalysts [27,28,29]. These studies show that the strong synergism between metal (e.g., Co) and nitrogen-doped carbon (N-C) can produce numerous Co-N-C active sites towards the ORR, meanwhile, the graphitized structure and heteroatom-doped carbon obtained after high-temperature carbonization facilitates the electrochemical process [26,29]. However, MOF derivatives with a single metal component are often difficult to achieve high-performance catalysis due to inherent limitations. For instance, ZIF-8-derived materials have been well-documented to possess high N content and large surface area, but with a limited degree of graphitization and lack of critical TM-N_x_ sites; when ZIF-67 is used as the precursor, a large amount of Co agglomeration will be formed in the obtained catalyst owing to the high content of Co [30,31]. Although considerable efforts have been devoted to bimetallic MOFs, the catalysts previously reported are mostly confined to the form of nanoparticles, and the OER performance is often insufficient, which severely restricts their practical applications in ZABs [27]. Consequently, it is necessary to explore new morphologies as well as self-supporting growth strategies for MOFs, while coupling them with catalysts having excellent OER activity, to counter these issues.

Lately, layered double hydroxides (LDHs), especially multi-metal LDH (e.g., CoFe, NiFe, CoNiFe), have emerged as attractive OER catalysts due to the remarkable catalytic performance, unique two-dimensional (2D) structure, and affordable [32,33]. In continuous research, CoFe-LDH electrocatalysts were found to display great potential for OER due to the synergistic effect between Co and Fe ions, which strengthens the breaking of the Co-O bond and the formation of O-O bond [34,35]. Nonetheless, the low utilization efficiency of active sites and poor electronic conductivity results in performance that does not meet the needs of the application [36]. Combining LDHs with three-dimensional (3D) conductive bones with good electronic conductivity, such as nickel/copper foam, graphene, carbon nanotubes, and carbon cloth (CC), has been shown to effectively promote electron transfer and electrolyte penetration, thereby addressing these shortcomings [37]. Recently, Song et al. proposed a 3D heterostructure consisting of NiSe and CoFe-LDH supported on the nickel foam for water splitting, and the obtained catalyst presents excellent OER catalytic performance [38]. Feng’s group integrated NiCo_2_S_4_ and Mo-doped CoFe-LDH on a nickel foam to gain a hybrid catalyst with high OER activity and durability for the application of industrial electrolytic water [39]. However, their ORR performance is still unsatisfactory, making it impossible to apply them as a bifunctional catalyst in ZABs. Thus, some additional efforts are needed to further improve their electrochemical performance, including the formation of heterojunction and compounding with other ORR functional materials [40,41]. These approaches to building heterostructural composites may achieve synergistic effects while creating new electrocatalytic sites through an interface and electronic engineering, which are beneficial for multifunctional catalysis [37]. For example, Hu et al. constructed an OER/ORR bifunctional catalyst with effective interfacial interactions, employing Co_3_O_4_ as the core and NiFe-LDH as the shell [42]. Considering the advantages of MOF derivatives, exploring the coupling of LDHs on the surface of MOF-based catalysts via interfacial engineering seems to be a promising strategy to develop efficient bifunctional electrocatalysts for rechargeable ZABs.

Inspired by the above, we hereby report a high-performance flexible self-supporting catalyst with a 3D hierarchical structure, composed of 3D leaf-like bimetallic MOFs-derived hybrid carbon (Co@NC) nanoflake nanoarrays, 2D ultrathin CoFe-LDH nanosheets, and 1D carbon fibers, for rechargeable and flexible ZABs. Through the cooperation of 3D interconnection open network structure and effective interface electron transfer effect, the prepared catalyst (CC/Co@NC/CoFe-LDH-700) can ensure sufficient active sites, improve the electronic conductivity of CoFe-LDH nanosheets, and achieve the balance of OER and ORR activity. As expected, CC/Co@NC/CoFe-LDH-700 catalyst displays superior OER performance with low overpotential (224 mV at 10 mA cm^−2^, 298 mV at 50 mA cm^−2^), and exhibits excellent ORR properties comparable to commercial Pt/C. When employed as an air cathode, the CC/Co@NC/CoFe-LDH-700-based liquid ZAB delivers satisfactory cell performance with a high open-circuit voltage of 1.47 V, a remarkable peak power density of 129.3 mW cm^−2^, and a large specific capacity of 710.7 mAh g^−1^_Zn_. More importantly, all-solid-state flexible ZABs assembled by CC/Co@NC/CoFe-LDH-700 self-supporting cathode demonstrate excellent rechargeable performance, exceptional cycling stability, and reliable flexibility, implying great potential in flexible wearable energy storage devices.

## 2. Materials and Methods

### 2.1. Materials and Chemicals

Zinc nitrate hexahydrate (Zn(NO_3_)_2_·6H_2_O), zinc acetate (Zn(CH_3_COO)_2_), ferric nitrate nonahydrate (Fe(NO_3_)_3_·9H_2_O), cobalt nitrate hexahydrate (Co(NO_3_)_2_·6H_2_O), potassium hydroxide (KOH), nitric acid (HNO_3_), and ethanol were all offered by Sinopharm Chemical Reagent Co., Ltd., Shanghai, China. Polyvinylalcohol (PVA, M_w_ = 195,000), 2-methylimidazole (2-MeIM), and 5.0 wt% Nafion were purchased from Shanghai Aladdin Chemical Co., Ltd., Shanghai, China. Carbon cloth (CC, W0S1009) was supplied by CeTech Co., Ltd., Taichung City, Taiwan. Commercial ruthenium oxide (RuO_2_) and 20 wt% Pt/C were obtained from MERYER Co., Ltd. (Shanghai, China) and Sigma-Aldrich (St. Louis, MO, USA), respectively.

### 2.2. Synthesis of Electrocatalysts

Synthesis of bimetallic MOF-derived Co@NC nanoflakes on CC: First, the carbon cloth (CC) substrate was immersed into the HNO_3_ and pre-treated at 120 °C for 3 h. Then, 2D bimetallic MOF (Zn, Co-MOF) precursors were grown on the surface of carbon fibers via a simple solution reaction. Briefly, the acidified CC was immersed in a mixed solution of 0.05 M Co(NO_3_)_2_·6H_2_O, 0.05 M Zn(NO_3_)_2_·6H_2_O, and 0.3 M 2-MeIM, and stood at room temperature for 12 h. After washing with deionized water and ethanol, followed by drying at 60 °C, the purple Zn, Co-MOF precursors were grown on CC (CC/Zn, Co-MOF). Then, the as-prepared CC/Zn, Co-MOF samples were placed in a quartz boat and annealed in Ar atmosphere at 900 °C with a ramping rate of 5 °C min^−1^ for 2 h. Finally, the black Co@NC nanoflakes on CC (CC/Co@NC) were gained after natural cooling to room temperature.

Synthesis of 2D CoFe-LDH nanosheets on CC/Co@NC: The 2D CoFe-LDH nanosheets were further anchored on the as-prepared Co@NC nanoflakes (CC/Co@NC/CoFe-LDH) using a rapid electrodeposition process. Typically, the electrodeposition electrolyte was composed of 0.02 M Co(NO_3_)_2_·6H_2_O and 0.02 M Fe(NO_3_)_3_·9H_2_O, meanwhile, the CC/Co@NC sample, Ag/AgCl electrode, and Pt wire served as the working, reference, and counter electrode, respectively. The electrodeposition of CoFe-LDH nanosheets was carried out at an applied potential of -1.0 V vs. Ag/AgCl with different deposition times of 500, 700, and 900 s, which was denoted as CC/Co@NC/CoFe-LDH-500, CC/Co@NC/CoFe-LDH-700, CC/Co@NC/CoFe-LDH-900, respectively. As a comparison, the bare CC was electrodeposited for 700 s under the same condition to prepare CC/CoFe-LDH-700; the CC/Co@NC was electrodeposited for 700 s in the electrolyte of only 0.02 M Co(NO_3_)_2_·6H_2_O or only 0.02 M Fe(NO_3_)_3_·9H_2_O to prepare CC/Co@NC/Co_x_(OH)_y_-700 and CC/Co@NC/Fe_x_(OH)_y_-700, respectively. Furthermore, the dispersion solution of RuO_2_ or 20 wt% Pt/C with 30 mL of 5 wt% Nafion, 485 mL of ethanol, and 485 mL of water was dropped evenly on the CC to obtain the reference sample with the same loading as CC/Co@NC/CoFe-LDH-700.

### 2.3. Material Characterization

The micromorphologies of as-obtained catalysts were characterized by scanning electron microscope (SEM, Zeiss Gemini 300), energy dispersive X-ray spectroscopy (EDS, Super-X), and transmission electron microscopy (TEM, F200X), respectively. A Quantachrome-EVO was employed to measure nitrogen adsorption-desorption isotherms at 77 K. The specific surface areas and pore distribution of samples were obtained by Brunauer-Emmett-Teller (BET) measurements. The X-ray diffraction (XRD) patterns were conducted on a Rigaku x-ray diffractometer (2550VB). X-ray photoelectron spectroscopy (XPS) was collected by Thermo Scientific K-Alpha with a monochromatic Al Kα source.

### 2.4. Electrochemical Measurements

The OER and ORR catalytic performance of samples were tested on CHI 760e electrochemical workstation using a standard three-electrode system. Hg/HgO and graphite rod acted as reference electrode and counter electrode, respectively. The potentials were calibrated to a reversible hydrogen electrode (RHE) by the formula of E(RHE) = E(Hg/HgO) + 0.059 × pH + 0.098. For OER test, the sample with a size of 1 × 1 cm^2^ was directly used as the working electrode. Linear scanning voltammetry (LSV) curves were tested at a scan rate of 2 mV·s^−1^ without iR compensation. Electrochemical impedance spectroscopy (EIS) was performed from 0.01 Hz to 1000 kHz with an amplitude of 5 mV. Double-layer capacitance (C_dl_) values were calculated by cyclic voltammetry (CV) curves at different scan rates (v) in the non-Faradic potential range through the formula: i_c_ = v × C_dl_, in which i_c_ represents charging current obtained at different scan rates (20, 40, 60, 80 and 100 mV·s^−1^). The long-time and accelerated stability were evaluated by the chronopotentiometry at a constant current of 50 mA cm^−2^ for 50 h, and the CVs at a scan rate of 100 mV·s^−1^ for 1000 cycles, respectively. For the ORR test, an acrylic tape (3 M) without electric conductivity will be used to fix the prepared sample (such as CC/Co@NC/CoFe-LDH-700) to the tip of a rotating disk electrode (RDE, 4 mm in diameter) to collect electric current from the working electrode, with a working area of 0.1256 cm^−2^. CVs were carried out in N_2_/O_2−_ saturated KOH solution with a scan rate of 50 mV·s^−1^ until the current was stabilized. LSVs were measured at a scan rate of 10 mV·s^−1^ with rotation rates ranging from 400 to 2025 rpm. The electron transfer number (n) was determined by Koutecky-Levich (K-L) equation, and the details were provided in the Supporting Information (S1). The long-term ORR stability of as-prepared catalysts was measured at 0.4 V vs. RHE for 20,000 s at a rotation rate of 1600 rpm via chronoamperometric response.

### 2.5. Liquid and Flexible Solid Zn-Air Battery Assembly

The liquid Zn-air battery was assembled with polished Zn foil and the CC/Co@NC/CoFe-LDH-700 (1 mg cm^−2^) as anode and cathode, respectively; meanwhile, the 6.0 M KOH solution containing 0.2 M Zn(CH_3_COO)_2_ was used as the electrolyte. The open circuit voltage and charge-discharge polarization curve were recorded using CHI 760e electrochemical workstation. The specific capacity was examined by the LAND BT2018R multi-channel battery testing system. The sandwich-like flexible solid-state Zn-air battery was assembled to assess the potential applications of the resulting catalysts. First, an alkaline gel electrolyte was fabricated as follows: 2 g of PVA powder was added to 20 mL of 6.0 M KOH solution containing 0.2 M Zn(CH_3_COO)_2_ and stirred at 90 °C until the solution was homogeneous; the above solution was poured into a square glass container to form a thin film of about 1 mm, which was frozen in a refrigerator overnight and thawed at room temperature. Then, the self-supporting catalyst and polished zinc foil were used as air electrode and anode respectively, which were connected to different sides of the solid electrolyte, and nickel foam served as a current collector. Finally, a flexible solid-state Zn-air battery was packaged with an aluminum-plastic film, and characterized by CHI 760e workstation and LAND BT2018R multi-channel battery testing system.

## 3. Results

**Figure 1:**
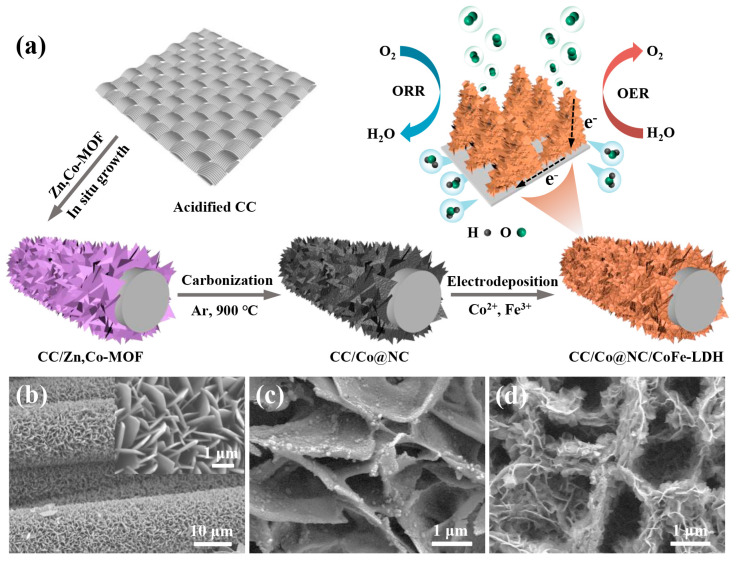
(**a**) Schematic diagram depicting the synthesis process of CC/Co@NC/CoFe-LDH. SEM images of (**b**) CC/Zn, Co-MOF, (**c**) CC/Co@NC, and (**d**) CC/Co@NC/CoFe-LDH-700.

Taking advantage of superior conductivity, large surface area, excellent flexibility, and sufficient mechanical strength, commercial carbon cloth (CC) was determined as the conductive substrate of a self-supporting electrode to meet the needs of flexible electronic devices. Figure 1a schematically depicts the preparation procedure of 3D hierarchical heterostructure Co@NC/CoFe-LDH on CC substrate. First, the 2D bimetallic Zn, Co-MOF precursors were in-situ grown on the surface of carbon fibers via a facile solution reaction, and then converted into Co nanoparticle-embedded nitrogen-doped porous carbon (Co@NC) nanoflake arrays by high-temperature carbonization. It should be mentioned that the existence of Zn species in the MOF could bring about the spatial isolation for Co species, which helps to inhibit the aggregation of Co nanoparticles during pyrolysis [30]. Finally, the ultrathin CoFe-LDH nanosheets were loaded onto Co@NC nanoflakes by electrodeposition, constructing a 3D hierarchical CC/Co@NC/CoFe-LDH-700 self-supporting electrode. There is no doubt that this open porous and interconnected network structure, formed by the longitudinal extension of Co@NC nanoflakes and the lateral extension of CoFe-LDH nanosheets, can provide abundant active sites for redox reactions as well as facilitate the rapid transport of substances (such as O_2_, H_2_O and OH^−^) [43].

Taking advantage of superior conductivity, large surface area, excellent flexibility, and sufficient mechanical strength, commercial carbon cloth (CC) was determined as the conductive substrate of self-supporting electrodes to meet the needs of flexible electronic devices. Figure 1a schematically depicts the preparation procedure of 3D hierarchical heterostructure Co@NC/CoFe-LDH on CC substrate. First, the 2D bimetallic Zn, Co-MOF precursors were in-situ grown on the surface of carbon fibers via a facile solution reaction, and then converted into Co nanoparticle-embedded nitrogen-doped porous carbon (Co@NC) nanoflake arrays by high-temperature carbonization. It should be mentioned that the existence of Zn species in the MOF could bring about the spatial isolation for Co species, which helps to inhibit the aggregation of Co nanoparticles during pyrolysis [30]. Finally, the ultrathin CoFe-LDH nanosheets were loaded onto Co@NC nanoflakes by electrodeposition, constructing a 3D hierarchical CC/Co@NC/CoFe-LDH-700 self-supporting electrode. There is no doubt that this open porous and interconnected network structure formed by the longitudinal extension of Co@NC nanoflakes and the lateral extension of CoFe-LDH nanosheets can provide abundant active sites for redox reactions as well as facilitate the rapid transport of substances (such as O_2_, H_2_O, and OH^−^) [43].

The scanning electron microscope (SEM) was first employed to investigate the microcosmic appearance of the as-obtained samples. Figure 1b displays the SEM images of Zn, Co-MOF precursors on CC substrate. Compared with the smooth surface of bare CC (Figure S1), numerous MOF nanoflakes were vertically arranged on the surface of carbon fiber, forming an interconnected porous 3D network (inset of Figure 1b). After carbonization, the bimetallic Zn, Co-MOF was transformed into Co nanoparticle-embedded nitrogen-doped porous carbon, and the previous nanoflake array structure was maintained perfectly (Figure 1c), offering a stable scaffold for the growth of CoFe-LDH. As expected, ultrathin CoFe-LDH nanosheets were uniformly anchored on the surface of interconnected Co@NC nanosheets (Figure 1d), and the heterojunction structure of Co@NC/CoFe-LDH was successfully constructed. In addition, the N_2_ adsorption-desorption isotherm of CC/Co@NC/CoFe-LDH-700, in Figure S2, exhibits a typical IV characteristic with a hysteresis loop, showing the mesoporous features, which correspond well to the pore size distribution. This highly open and porous structure endows the Co@NC/CoFe-LDH-700 catalyst with a large specific surface area of 26.4 m^2^ g^−1^, comparable to or larger than that of the recently reported catalysts on CC (Table S1), which can provide sufficient accessible reaction sites and fast mass transport for the electrocatalytic process [44]. 

**Figure 2:**
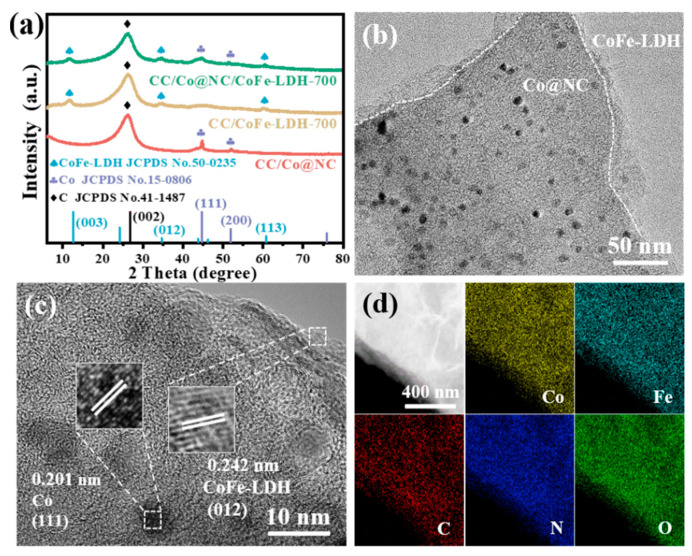
(**a**) XRD patterns of related samples. (**b**) TEM and (**c**) HRTEM images of CC/Co@NC/CoFe-LDH-700. (**d**) Elemental mapping images of CC/Co@NC/CoFe-LDH-700.

The compositions and crystalline phases of CC/Co@NC, CC/CoFe-LDH-700, and CC/Co@NC/CoFe-LDH-700 were identified using XRD measurements. As presented in Figure 2a, the characteristic peak located at about 26.5° belongs to the (002) plane of graphitic carbon from Co@NC and CC substrate (JCPDS No.41-1487) [45]. Meanwhile, the diffraction peaks appearing at 44.2° and 51.5° in CC/Co@NC match well with the (111) and (200) planes of metallic Co (JCPDS No.15-0806), deriving from the Co nanoparticles obtained by the carbonization of the Zn, Co-MOFs [27]. For the CC/Co@NC/CoFe-LDH-700, the additional peaks at 11.6°, 34.0°, and 60.5° correspond to the (003), (012), and (113) planes of CoFe-LDH, while the similar peaks can be observed in CC/CoFe-LDH-700 [33]. TEM image verifies that Co nanoparticles with an average size of about 10 nm and ultrathin CoFe-LDH nanosheets were decorated on the surface of MOF-derived carbon nanoflakes (Figure 2b). Especially, the clear lattice fringes with distances of 0.201 and 0.242 nm are observed in the high-resolution TEM (HRTEM) image (Figure 2c), corresponding well to Co (111) and CoFe-LDH (012) planes, respectively [41,46]. The co-existence of Co@NC and CoFe-LDH enables the good ORR and OER activities of catalysts, while the effective contact of the two phases is beneficial to enhance electron transport and catalytic stability through a “synergistic effect” [40]. Moreover, the agglomeration of Co is found to occur when Co-MOF was carbonized at high temperature, resulting in large Co nanoparticles with uneven distribution (Figure S3), which is in sharp contrast to the small and uniformly dispersed nanoparticles derived from bimetallic MOFs. The results indicate that the steric barrier effect of Zn species can effectively suppress Co sintering during pyrolysis [31]. In addition, the elemental mapping images in Figure 2d illustrate the homogeneous distribution of Co, Fe, C, N, and O elements in the detection region of the heterostructure Co@NC/CoFe-LDH.

**Figure 3:**
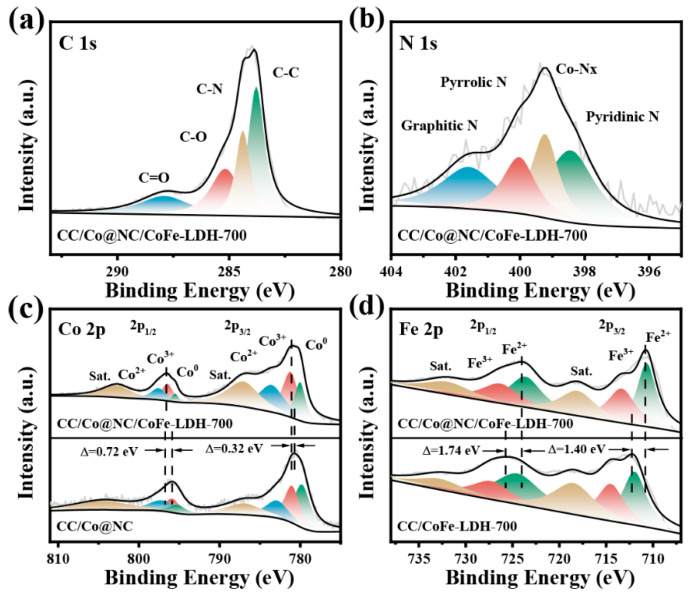
High-resolution XPS spectra of (**a**) C 1s, (**b**) N 1s, (**c**) Co 2p, and (**d**) Fe 2p for related samples.

The valance state and electron interaction of the constituting elements in CC/Co@NC/CoFe-LDH-700 were further investigated by XPS. It can be seen from the XPS full spectra in Figure S4 that there are mainly C, N, Co, Fe, and O elements in the CC/Co@NC/CoFe-LDH-700, agreeing with the element scanning results [47,48,49]. In Figure 3a, the high-resolution C 1s spectra was deconvolved into four different functional carbons, corresponding to C-C at 283.8 eV, C-N at 284.4 eV, C-O at 285.2 eV, and C=O at 287.9 eV, respectively. The C-N bond suggests the successful doping of N atoms in the carbon framework, while the C-C bond indicates the presence of graphitic carbon [11]. The N 1s spectra shown in Figure 3b was then divided into four kinds of N species, containing pyridinic N (398.4 eV), Co-N_x_ (399.2 eV), pyrrolic N (400.0 eV), and graphitic N (401.6 eV). Studies have shown that most of the N bonding configurations, except for N-oxide, contribute to the electrocatalytic activity, for example, pyridinic N boosts the adsorption of oxygen species, and graphitic N enhances the conductivity [50]. In particular, Co-N_x_ can serve as a highly active center for ORR in electrocatalysts [51]. Figure 3c presents the Co 2p spectra for CC/Co@NC/CoFe-LDH-700 and CC/Co@NC, two prominent peaks located at 780.8 eV and 796.5 eV correspond to Co 2p_3/2_ and Co 2p_1/2_, while the subsequent peaks at 787.0 eV and 802.7 eV can be attributed to the satellite peaks (identified as Sat.) of Co 2p_3/2_ and Co 2p_1/2_, respectively, implying the co-existence of the Co^2+^ and Co^3+^ species [29]. Moreover, the deconvoluted peaks of 779.9 eV and 795.5 eV are assigned to Co^0^, confirming the presence of metal Co, which is consistent with the results of XRD and TEM [52]. The Fe 2p spectrum of CC/Co@NC/CoFe-LDH-700 in Figure 3d shows Fe 2p_3/2_ and Fe 2p_1/2_ characteristic peaks at 710.8 eV and 724.1 eV, which can be fitted into four peaks of Fe^3+^ (713.3 eV and 726.3 eV) and Fe^2+^ (710.6 eV and 723.6 eV), accompanied by two satellite peaks (718.0 eV and 731.9 eV) [38]. Impressively, it can be seen that Co 2p in CC/Co@NC/CoFe-LDH-700 has a significant positive shift compared with CC/Co@NC, meanwhile, Fe 2p in CC/Co@NC/CoFe-LDH-700 shifts negatively relative to CC/CoFe-LDH-700, which is caused by interfacial interaction between the Co@NC and CoFe-LDH [53]. It has been demonstrated that this strong interaction results from the partial electron transfer from Co@NC to CoFe-LDH, which helps to regulate the redox behavior of Co and Fe cations, thus generating the active phase in the electrochemical process [43,54]. In addition, the interfacial electron interaction between the Co@NC and CoFe-LDH is essential for rapid charge transfer, which can be verified by the follow-up electrochemical tests. 

**Figure 4:**
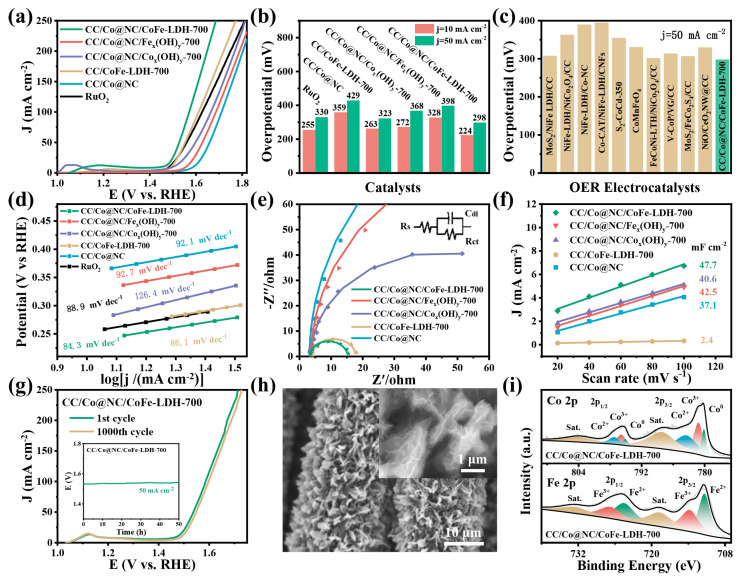
OER performance of the as-prepared samples. (**a**) LSV Polarization curves at a scan rate of 2 mV/s. (**b**) Comparison of overpotentials at the current density of 10 mA cm^−2^ (η_10_) and 50 mA cm^−2^ (η_50_). (**c**) Comparison of overpotentials at the current density of 50 mA cm^−2^ between CC/Co@NC/CoFe-LDH-700 and other recently reported OER electrocatalysts. (**d**) Tafel plots. (**e**) Nyquist plots and the inset shows the equivalent circuit. (**f**) The C_dl_ obtained from the CV curves. (**g**) Polarization LSV curves before and after 1000 CV cycles, and the inset shows a chronopotentiometry curve at a constant current density of 50 mA cm^−2^ for 50 h. (**h**) SEM images and (**i**) XPS spectra of Co 2p and Fe 2p for CC/Co@NC/CoFe-LDH-700 after stability measurement.

The electrocatalytic OER performance of CC/Co@NC/CoFe-LDH-700 and other contrast samples was first evaluated by LSV measurements in 1.0 M KOH solution. As seen in Figure 4a,b, CC/Co@NC/CoFe-LDH-700 exhibits excellent OER catalytic activity, requiring only the overpotential of 224 and 298 mV to obtain the current density of 10 and 50 mA cm^−2^ (η_10_ = 224 mV, η_50_ = 298 mV) without *IR* correction, which are smaller than these of CC/Co@NC (359, 429 mV), CC/CoFe-LDH-700 (263, 323 mV), CC/Co@NC/Co_x_(OH)_y_-700 (272, 368 mV), CC/Co@NC/Fe_x_(OH)_y_-700 (328, 398 mV), and even commercial RuO_2_ (255, 330 mV), respectively. It is worth noting that the effect of CoFe-LDH nanosheets with different electrodeposition times on the OER performance of CC/Co@NC/CoFe-LDH was also investigated, and it was found that the catalyst with an electrodeposition time of 700 s shows the lowest overpotential at 50 mA cm^−2^ (Figure S5). This is because a short electrodeposition time will result in the low loading of CoFe-LDH nanosheets, while a long time will lead to the accumulation of CoFe-LDH nanosheets and hinder mass transport [41]. Only proper electrodeposition time can ensure abundant catalytic active sites and efficient mass exchange in the catalyst. Moreover, the OER catalytic activity (overpotentials at the current density of 50 mA cm^−2^) of CC/Co@NC/CoFe-LDH-700 is superior to most reported conventional LDHs and many other similar recently reported high-performance OER catalysts (Figure 4c and Table S2).

Figure 4d illustrates the Tafel slopes calculated from the LSV curves using the formula of η = b log j + c, where η represents the overpotential, b represents the slope, j represents the current density, and c represents the exchange current. Clearly, CC/Co@NC/CoFe-LDH-700 presents the smallest Tafel slope of 84.3 mV dec^−1^, which outperforms CC/Co@NC (92.1 mV dec^−1^), CC/CoFe-LDH-700 (86.1 mV dec^−1^), CC/Co@NC/Co_x_(OH)_y_-700 (126.4 mV dec^−1^), CC/Co@NC/Fe_x_(OH)_y_-700 (92.7 mV dec^−1^), and RuO_2_ (88.9 mV dec^−1^), suggesting a more favorable OER reaction kinetics. The EIS was employed to further study the interface reactions and electrode kinetics, and the Nyquist plots fitted with the equivalent circuit are shown in Figure 4e, in which R_s_, C_dl_, and R_ct_ represent solution resistance, capacitance, and charge transfer resistance. The charge transfer resistance (R_ct_) is closely related to the electrocatalytic kinetics, and a lower R_ct_ value means a faster kinetic reaction. Compared with CC/Co@NC, CC/CoFe-LDH-700, CC/Co@NC/Co_x_(OH)_y_-700, and CC/Co@NC/Fe_x_(OH)_y_-700, the CC/Co@NC/CoFe-LDH-700 possesses the smallest R_ct_ value, indicating a fast charge transfer ability. The inseparable growth of CoFe-LDH nanosheets on the surface of Co@NC nanoflakes can effectively accelerate the charge transfer and reduce the R_ct_ through the synergistic effect, which is consistent with the results of low overpotential and small Tafel slope.

Electrochemically active surface area (ECSA) is one of the important parameters to evaluate the intrinsic activity of catalysts, which is directly proportional to the C_dl_, and a larger ECSA means more exposed active sites for electrocatalytic reactions. As revealed in Figure 4f, the C_dl_ (47.7 mF cm^−2^) of CC/Co@NC/CoFe-LDH-700 was obtained from the CVs in the non-faradaic potential region (Figure S6) which is higher than that of CC/Co@NC (37.1 mF cm^−2^), CC/CoFe-LDH-700 (2.4 mF cm^−2^), CC/Co@NC/Co_x_(OH)_y_-700 (40.6 mF cm^−2^), and CC/Co@NC/Fe_x_(OH)_y_-700 (42.5 mF cm^−2^). This result implies that the CC/Co@NC/CoFe-LDH-700 inherits the large specific surface area of the MOF-derived catalyst, meanwhile, the 3D hierarchical heterojunction structure effectively improves the exposure of active sites, thereby enhancing the OER activity. Moreover, long-term stability is another important criterion in the practical application of electrocatalysis. From the accelerated stability of Figure 4g, it can be observed that the OER polarization curves of CC/Co@NC/CoFe-LDH-700 undergo negligible decay after 1000 cycles, manifesting good stability. Beyond that, the CC/Co@NC/CoFe-LDH-700 also kept almost constant potential after continuous oxygen evolution at 50 mA cm^−2^ for 50 h (inset of Figure 4g), and the morphology and composition of the catalyst was still well-maintained after long-term OER reaction (Figure 4h,i), demonstrating superior durability and structural stability.

**Figure 5:**
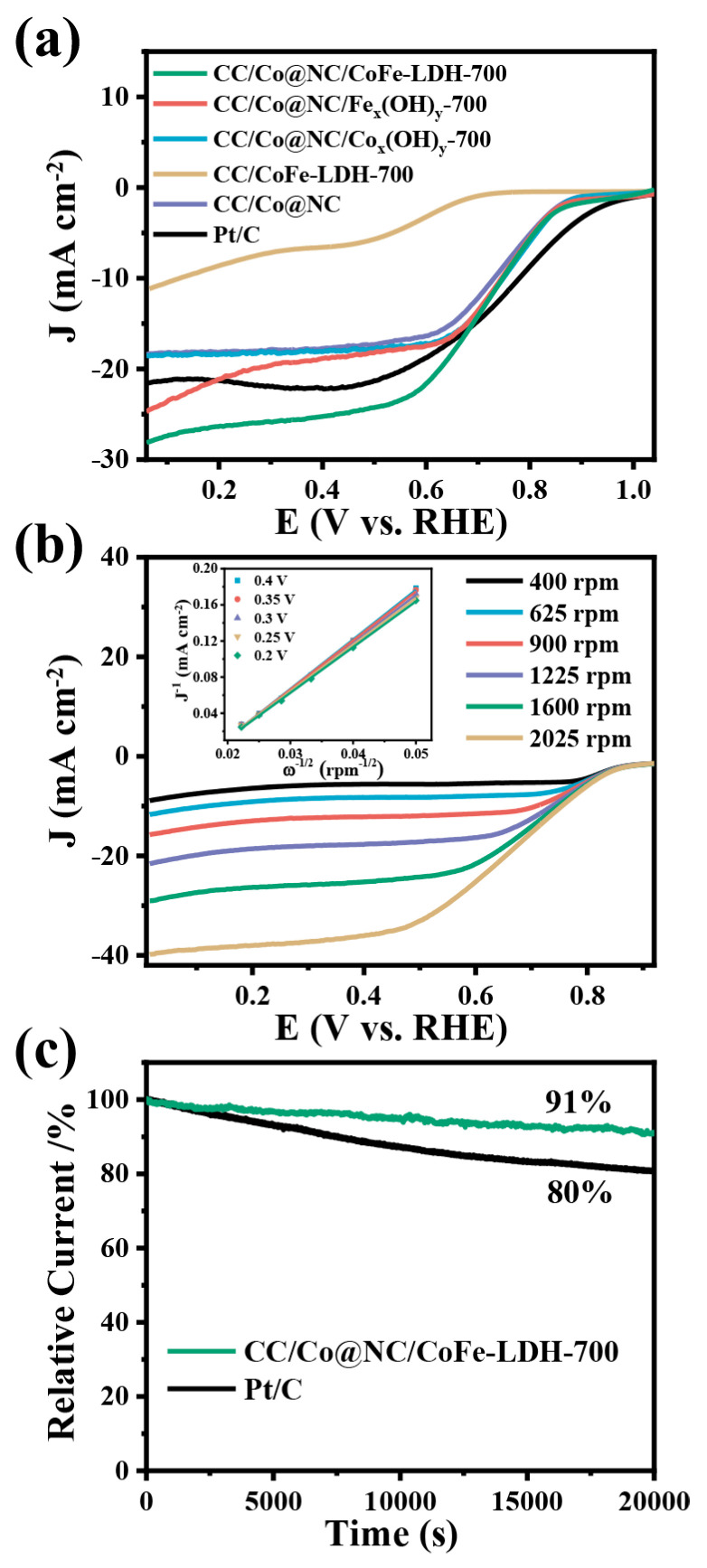
(**a**) ORR polarization curves of related samples at a rotation speed of 1600 rpm and a scan rate of 10 mV/s. (**b**) LSV curves of CC/Co@NC/CoFe-LDH-700 at different rotation speeds, and the inset is the corresponding K–L plots at various potentials. (**c**) Normalized chronoamperometric curves of CC/Co@NC/CoFe-LDH-700 and Pt/C at 0.4 V.

In addition to OER performance, the ORR activity and stability of CC/Co@NC/CoFe-LDH-700 was also assessed in 0.1 M KOH solution. The CV tests in Figure S7 show that the CC/Co@NC/CoFe-LDH-700 exhibits a typical oxygen reduction peak in the O_2_-saturated electrolyte, whereas this reduction peak is not observed in the N_2_-saturated solution, signifying remarkable ORR performance. The same result can be obtained by the rotating disk electrode (RDE) measurements, as presented in Figure 5a. Compared with other comparative samples, the CC/Co@NC/CoFe-LDH-700 electrode displays a higher onset potential (E_0_, 0.83 V), half-wave potential (E_1/2_, 0.69 V) and larger limiting current density (*J*_lim_, 29.0 mA cm^−2^), even comparable to Pt/C (E_0_ = 0.92 V, E_1/2_ = 0.76 V and *J*_lim_ = 21.3 mA cm^−2^) and recently reported catalyst on the carbon cloth substrate (Table S3). This excellent ORR performance is mainly attributed to the active site provided by Co@NC and the synergistic effect with CoFe-LDH interaction, while the presence of Zn species in MOF contributes to the uniform distribution of Co nanoparticles and effectively promotes the ORR performance (Figure S8) [30,31]. Similarly, we also explored the influence of different electrodeposition times (500, 700, and 900 s) on the ORR performance of CC/Co@NC/CoFe-LDH. It can be seen from Figure S9 that the electrodeposition time has little effect on the ORR performance. To gain insight into the ORR mechanism, the polarization LSV curves at different rotation speeds and corresponding K–L plots were carried out. As illustrated in Figure 5b, the limiting current density of CC/Co@NC/CoFe-LDH-700 is ascended as the rotation speed increases from 400 to 2025 rpm, indicating that the reaction process is kinetically controlled. The fitted K–L plots (the inset of Figure 5b) display a good linearity relationship, suggesting first-order reaction kinetics for dissolved oxygen. Furthermore, the average electron transfer number (n) of CC/Co@NC/CoFe-LDH-700 was calculated to be about four according to the K-L equation, demonstrating a nearly 4-electron transfer pathway in the ORR process. Chronoamperometric tests were then performed to investigate the stability (Figure 5c). The CC/Co@NC/CoFe-LDH-700 catalyst shows robust long-term durability with a current retention of 91% after 20,000 s of continuous operation, while that of Pt/C decreases to 80%.

**Figure 6:**
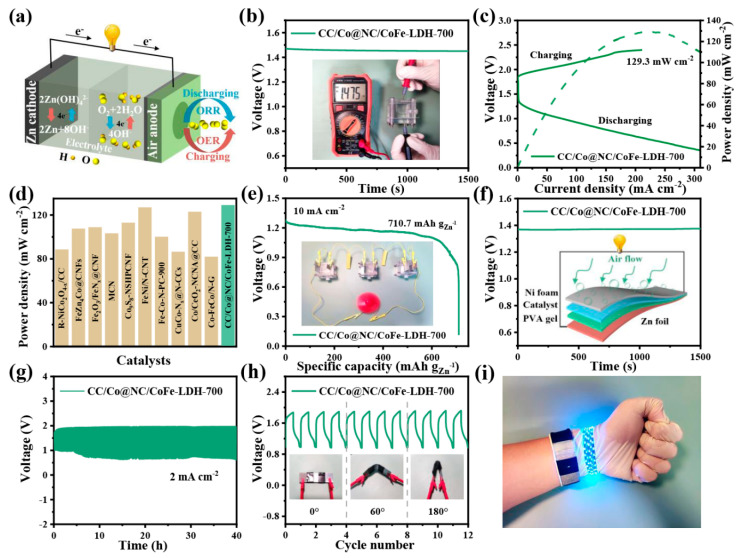
Electrochemical performance of ZABs with CC/Co@NC/CoFe-LDH-700 as the air cathode. (**a**) Schematic diagram of a liquid ZAB. (**b**) Open-circuit voltage measurement of the liquid ZAB. (**c**) Charge-discharge polarization curves and corresponding power density plot. (**d**) Performance comparison of liquid ZABs based on different air cathodes. (**e**) Specific capacity curve. (**f**) The open-circuit plot and the inset show a schematic illustration of a solid-state ZAB. (**g**) Galvanostatic cycling curve at 2 mA cm^−2^. (**h**) Cycling stability under different bending angles. (**i**) Photograph shows the application of wearable and flexible ZABs.

In view of the excellent OER/ORR bifunctional catalytic performance, the liquid Zn-air batteries (ZABs) were constructed with the CC/Co@NC/CoFe-LDH-700 catalyst as air cathode and the polished Zn foil as an anode to demonstrate its practical application (Figure 6a). As displayed in Figure 6b, the open-circuit voltage of CC/Co@NC/CoFe-LDH-700-driven liquid ZAB is about 1.47 V and keeps stable for 1500 s. From the typical charge and discharge polarization curves in Figure 6c, it can be seen that the CC/Co@NC/CoFe-LDH-700-based liquid ZAB exhibits a small charge-discharge voltage gap, suggesting superior rechargeability. A high peak power density of 129.3 mW cm^−2^ is obtained when the current density is 217.7 mA cm^−2^, which is superior to that of the recently reported self-supporting noble metal-free catalysts for Zn-air batteries (Figure 6d and Table S4). Moreover, the specific capacity normalized to the amount of zinc consumption reaches 710.7 mAh g^−1^_Zn_ at a current density of 10 mA cm^−2^, and the CC/Co@NC/CoFe-LDH-700-assembled liquid ZABs in series can easily power small red fan (Figure 6e). Inspired by the excellent flexibility of the CC/Co@NC/CoFe-LDH-700 catalyst and the exciting results of the corresponding liquid ZABs, the sandwich-like all-solid-state flexible ZABs were further assembled. Figure 6f reveals that the solid-state flexible ZAB can achieve a stable open circuit potential close to 1.4 V. Moreover, the CC/Co@NC/CoFe-LDH-700-based flexible ZAB displays outstanding stability for up to 40 h at a current density of 2 mA cm^−2^ (Figure 6g), and especially, the charge-discharge plateau remains stable at different folding angles (Figure 6h), indicating a long service life, satisfactory bendability, and mechanical stability. As a proof of concept, the CC/Co@NC/CoFe-LDH-700-driven all-solid-state flexible ZAB can successfully light up the LED strip and perform well throughout the bending process (Figure 6i), exhibiting considerable potential in wearable devices. 

## 4. Conclusions

In summary, we achieve simultaneous sufficient exposure of both OER/ORR active sites by in-situ electrodeposition of efficient OER catalysts (ultrathin CoFe-LDH nanosheets) on superior ORR catalysts (bimetallic MOF-derived Co@NC nanoflakes) with carbon cloth as support. Benefiting from the superior 3D hierarchical structure as well as the interface coupling interaction between the CoFe-LDH and Co@NC, the obtained catalyst exhibits excellent bifunctional catalytic properties, favorable reaction kinetics, and robust durability for OER/OER. Moreover, CC/Co@NC/CoFe-LDH-700 can be directly employed as an additive-free air cathode for liquid rechargeable ZABs without additional collectors, presenting a high open-circuit voltage of 1.47 V, a large peak power density of 129.3 mW cm^−2^ and a good specific capacity of 710.7 mAh g^−1^_Zn_. Impressively, the assembled all-solid-state flexible ZAB exhibits long cycling life of up to 40 h, satisfactory flexibility as well as good charge-discharge stability under different bending states, suggesting a good application prospect in portable and wearable electronics. This work offers valuable insights into the rational design of self-supporting bifunctional electrocatalysts for high-performance ZABs and related energy conversion equipment.

## Data Availability

Not applicable.

## Supplementary Materials

The following supporting information can be downloaded at: https://www.mdpi.com/article/10.3390/polym15030734/s1, Figure S1: SEM image of bare CC; Figure S2: N_2_ adsorption–desorption isotherm of CC/Co@NC/CoFe-LDH-700 and the corresponding pore-size distribution (inset); Figure S3: TEM image of CC/Co-MOF derived catalyst; Figure S4: XPS full spectra of CC/Co@NC/CoFe-LDH-700; Figure S5: LSV Polarization curves of CC/Co@NC/CoFe-LDH under different electrodeposition times; Figure S6: CV curves of (a) CC/Co@NC/CoFe-LDH-700, (b) CC/Co@NC/Co_x_(OH)_y_-700, (c) CC/Co@NC/Fe_x_(OH)_y_-700, (d) CC/CoFe-LDH-700 and (e) CC/Co@NC at different scan rates in the non-Faradaic potential; Figure S7: CV curves of CC/Co@NC/CoFe-LDH-700 in N_2_- and O_2_- saturated 0.1 M KOH at a scan rate of 50 mV·s^−1^; Figure S8: ORR polarization curves of CC/Zn, Co-MOF and CC/Co-MOF derived catalysts at a rotation speed of 1600 rpm; Figure S9: ORR polarization curves of CC/Co@NC/CoFe-LDH under different electrodeposition times at a rotation speed of 1600 rpm; Table S1: Comparison of the specific surface area of the CC/Co@NC/CoFe-LDH -700 with previously reported electrocatalysts; Table S2: Comparison of η50 between CC/Co@NC/CoFe-LDH-700 and other recently reported OER electrocatalysts; Table S3: Comparison of the ORR catalytic activity of the CC/Co@NC/CoFe-LDH-700 with previously reported electrocatalysts; Table S4: Comparison of the performance of liquid ZABs based on different air cathodes.
