# Supplementary material for: Interfacial Engineering of Leaf-like Bimetallic MOF-Based Co@NC Nanoarrays Coupled with Ultrathin CoFe-LDH Nanosheets for Rechargeable and Flexible Zn-Air Batteries
Source: Polymers (Basel). 2023 Jan 31;15(3):734. doi: 10.3390/polym15030734 (PMC9919106; doi:10.3390/polym15030734)
Supplement: Supplementary file 1 [file polymers-15-00734-s001.zip › polymers-2163449-supplementary.pdf]

Supporting Information

Interfacial engineering of leaf-like bimetallic MOF-based Co@NC nanoarrays coupled with ultrathin CoFe-LDH nanosheets for rechargeable and flexible Zn-air batteries

S1 Details for Calculation of the number of transferred electrons (n)

The number of electrons transferred (n) in the ORR process was calculated on the basis of the Koutecky-Levich equation:

$$\frac{1}{J} = \frac{1}{J_k} + \frac{1}{J_L} = \frac{1}{J_k} + \frac{1}{B\omega^{1/2}}$$

$$B = 0.2nFC_0{D_0}^{2/3}\,v^{-1/6}$$

where J, J_K, and J_L represent the measured current, kinetic current, and diffusion-limiting current, respectively. F is the Faraday constant (96,485 C mol^{-1}), ω is the electrode rotation speed, n is the number of electron transfer per oxygen molecule. D_0 is the diffusion coefficient of O_2 (1.9×10^{-5} cm^2 s^{-1}), C_0 is the bulk concentration of O_2 (1.2×10^{-6} mol cm^{-3}), and ν is the kinematic viscosity of the electrolyte (0.1 M KOH, 0.01 cm^2 s^{-1}) [1,2].

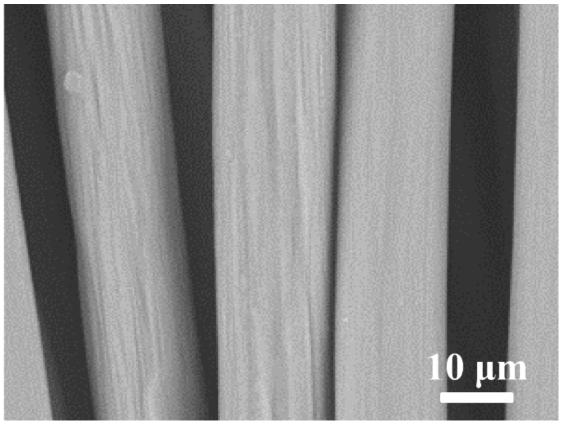

Figure S1. SEM image of bare CC.

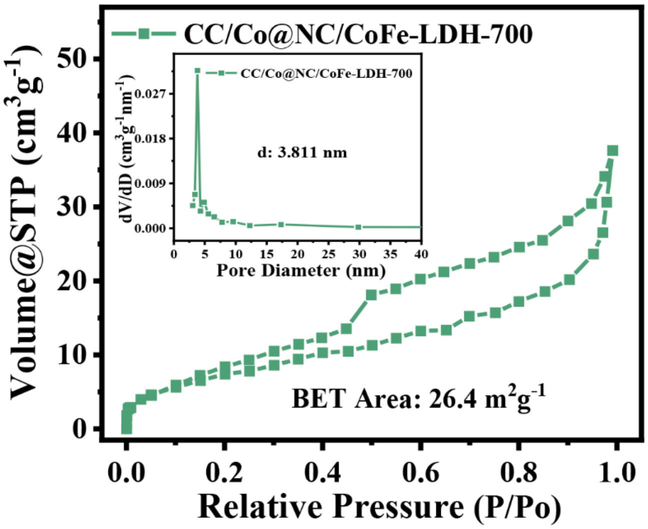

Figure S2. N_2 adsorption–desorption isotherm of CC/Co@NC/CoFe-LDH-700 and the corresponding pore-size distribution (inset).

Table S1. Comparison of the specific surface area of the CC/Co@NC/CoFe-LDH -700 with previously reported electrocatalysts.

Catalysts	Specific surface area (m^2 g^{-1})	References
CC/Co@NC/CoFe-LDH-700	26.4	This work
NiS_2/CC-180	10	[3]
$MnCo_2S_4$/CC	20.12	[4]
Mn-COPS-5%/CC	12.91	[5]
Nb-$CoSe_2$/CC	15.1	[6]
Ce-Co_9S_8@CC	15.3	[7]
$NiSe_2$@NiO	26.3	[8]
V-CoP_2/CC	22.8	[9]
Ni_3N−NiMoN/CC	13.5	[10]

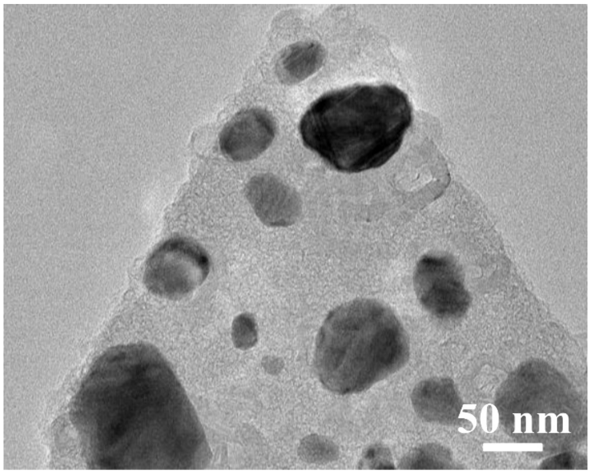

Figure S3. TEM image of CC/Co-MOF derived catalyst.

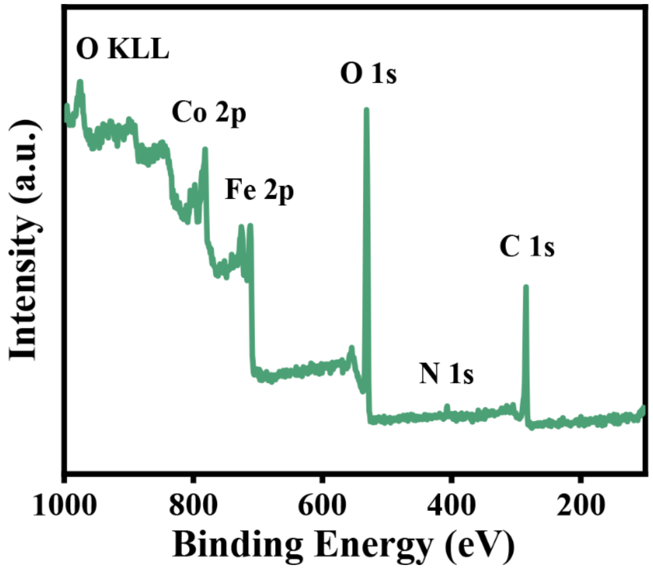

Figure S4. XPS full spectra of CC/Co@NC/CoFe-LDH-700.

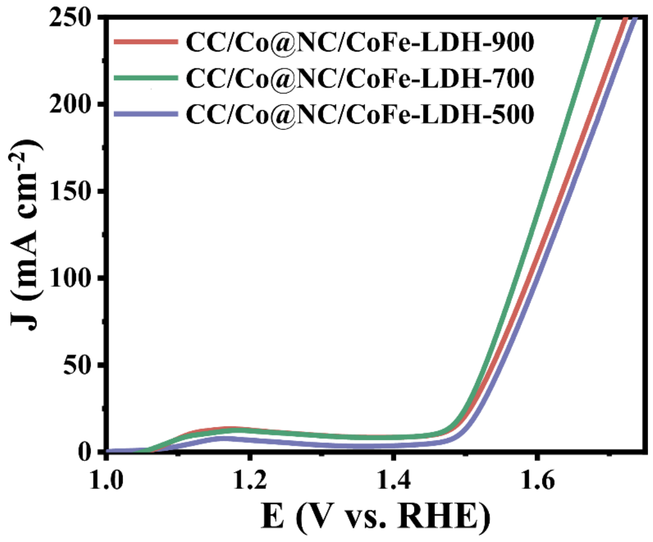

Figure S5. LSV Polarization curves of CC/Co@NC/CoFe-LDH under different electrodeposition times.

Table S2. Comparison of overpotentials at the current density of 50 mA cm^{-2} between CC/Co@NC/CoFe-LDH-700 and other recently reported OER electrocatalysts.

Catalysts	Overpotential (mV)	References
CC/Co@NC/CoFe-LDH-700	298	This work
MoS_2/NiFe LDH/CC	308	[11]
NiFe-LDH/$NiCo_2O_4$/CC	363	[12]
NiFe-LDH/Co-NC	390	[13]
Co-CAT/NiFe-LDH/CNFs	395	[14]
S_2-CoCd-350	355	[15]
$CoMnFeO_4$	331	[16]
FeCoNi-LTH/$NiCo_2O_4$/CC	302	[17]
V-CoP/VG/CC	314	[18]
MoS_2/$FeCo_2S_4$/CC	307	[19]
NiO/CeO_2NW@CC	330	[20]

MoS_2/NiS_2	348	[21]
W-Ni_2P NS/CC	382	[22]
NNO/CeO_2/NF	330	[23]
$Co(OH)_2$-$Ni(OH)_xS_y$ (Co:Ni = 9:1)	423	[24]

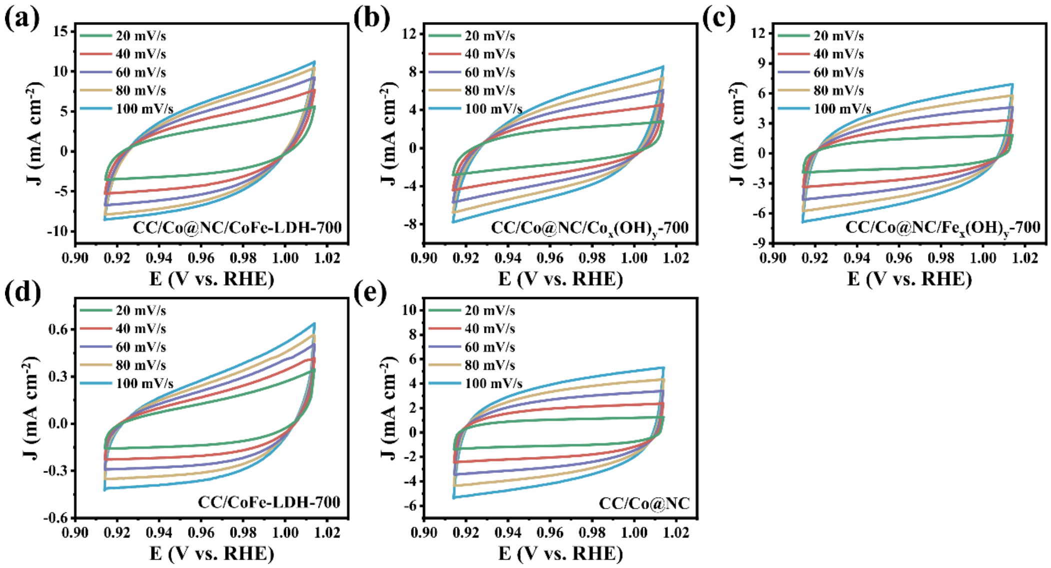

Figure S6. CV curves of (a) CC/Co@NC/CoFe-LDH-700, (b) CC/Co@NC/$Co_x(OH)_y$-700, (c) CC/Co@NC/$Fe_x(OH)_y$-700, (d) CC/CoFe-LDH-700 and (e) CC/Co@NC at different scan rates in the non-Faradaic potential.

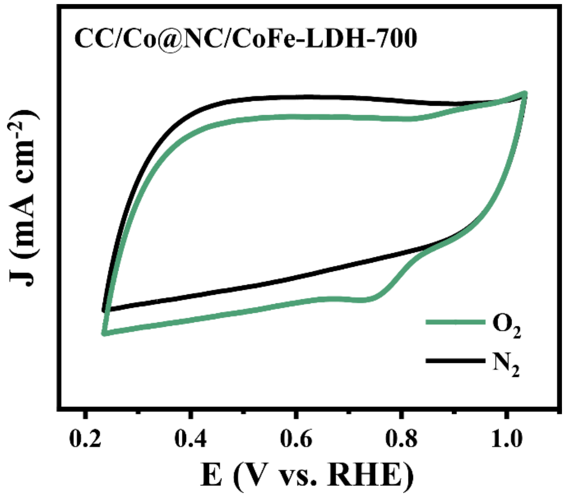

Figure S7. CV curves of CC/Co@NC/CoFe-LDH-700 in N_2- and O_2- saturated 0.1 M KOH at a scan rate of 50 mV s^{-1}.

Table S3. Comparison of the ORR catalytic activity of the CC/Co@NC/CoFe-LDH-700 with previously reported electrocatalysts.

Catalysts	$E_{1/2}$ (V)	J_{lim} (mA cm^{-2})	References
CC/Co@NC/CoFe-LDH-700	0.69	29.0	This work
Co-$CoO_χ$/N-C NSAs	0.83	10.3	[25]
NiFe/NCNF/CC	0.817	14.6	[26]
Co/CeO_2–NCNA@CC	0.77	22.8	[27]
Mn–Ni_3S_2/NF	0.63	4.9	[28]
m-NCO-NB	0.745	5.6	[29]
CoFe@NC/CC	0.75	5	[30]

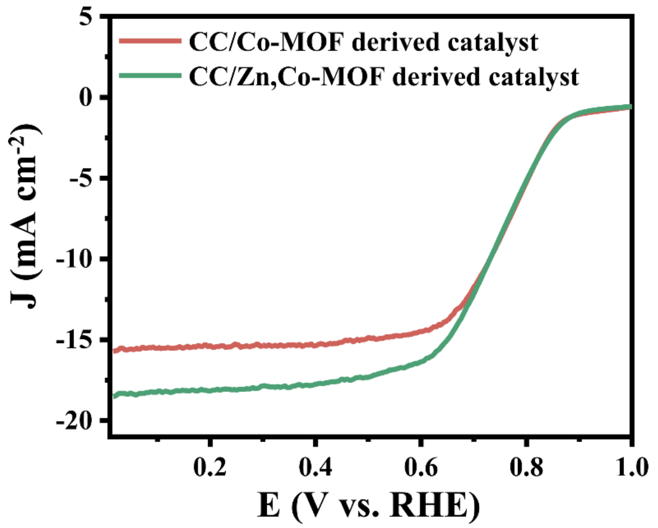

Figure S8. ORR polarization curves of CC/Zn, Co-MOF and CC/Co-MOF derived catalysts at a rotation speed of 1600 rpm.

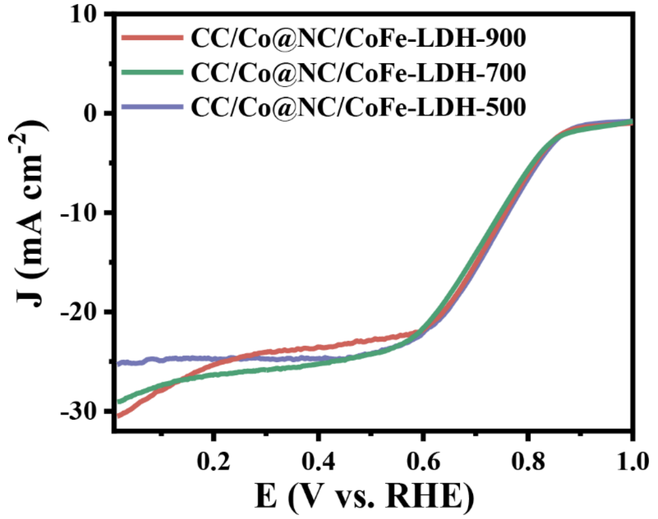

Figure S9. ORR polarization curves of CC/Co@NC/CoFe-LDH under different electrodeposition times at a rotation speed of 1600 rpm.

Table S4. Comparison of the performance of liquid ZABs based on different air cathodes.

Air cathodes	Power density (mW cm^{-2})	References
CC/Co@NC/CoFe-LDH-700	129.3	This work
R-$NiCo_2O_{4-x}$/CC	88.6	[31]
$FeZn_4Co$@CNFs	107.6	[32]
Fe_2O_3/FeN_x@CNF	108.9	[33]
MCN	103.3	[34]
Co_9S_8-NSHPCNF	113	[35]
FeNi/N-CNT	127	[36]
Fe-Co-N-PC-900	100.2	[37]
CuCo-N_x@N-CCs	86.5	[38]
Co/CeO_2-NCNA@CC	123	[27]
Co-FeCo/N-G	82	[39]
N, Co-CNTs	114	[40]
Co@NCW	47.5	[41]

References

1. Guo, J.X.;Chen, B.L.;Hao, Q.;Nie, J.;Ma, G.P. Pod-like structured Co/CoO_x nitrogen-doped carbon fibers as efficient oxygen reduction reaction electrocatalysts for Zn-air battery. *Appl. Surf. Sci.* **2018**, *456*, 959-966.
2. Zang, W.J.;Sumboja, A.;Ma, Y.Y.;Zhang, H.;Wu, Y.;Wu, S.S.;Wu, H.J.;Liu, Z.L.;Guan, C.;Wang, J.;Pennycook, S.J. Single co atoms anchored in porous N-doped carbon for efficient Zinc−air battery cathodes. *ACS Catal.* **2018**, *8*, 8961-8969.
3. Peng, X.;Yan, Y.J.;Xiong, S.J.;Miao, Y.P.;Wen, J.;Liu, Z.T.;Gao, B.;Hu, L.S.;Chu, P.K. Se-$NiSe_2$ hybrid nanosheet arrays with self-regulated elemental Se for efficient alkaline water splitting. *J. Mater. Sci. Technol.* **2022**, *118*, 136-143.
4. Ma, T.T.;Dai, Z.;Shen, X.R.;Jiao, Q.Z.;Zhao, Y.;Li, H.S.;Feng, C.H. Three-dimensional porous $MnCo_2S_4$ microrugby balls supported on carbon cloth for efficient oxygen evolution reaction. *ChemElectroChem* **2022**, *9*, e202200552.
5. Fan, J.Y.;Chen, M.Y.;Liu, B.X.;Xu, D.D.;Lin, Y.;Shi, N.E.;Liu, Y.;Dai, Z.H.;Bao, J.C.;Han, M.;Huang, W. Self-supported gold-silk-chrysanthemum-like superstructures arrays derived from Mn-doped CoPS nanowires with superhydrophilic and superaerophobic surface for enhanced oxygen evolution. *Adv. Mater. Interfaces* **2022**, *9*, 2200098.
6. Peng, Q.M.;Zhuang, X.L.;Wei, L.G.;Shi, L.Y.;Isimjan, T.T.;Hou, R.B.;Yang, X.L. Niobium-incorporated $CoSe_2$ nanothorns with electronic structural alterations for efficient alkaline oxygen evolution reaction at high current density. *ChemSusChem* **2022**, *15*, e202200827.
7. Liu, K.;Zhu, Z.Y.;Jiang, M.Q.;Li, L.C.;Ding, L.F.;Li, M.;Sun, D.M.;Yang, G.X.;Fu, G.T.;Tang, Y.W. Boosting electrocatalytic oxygen evolution over Ce−Co_9S_8 core–shell nanoneedle arrays by electronic and architectural dual engineering. *Chem. Eur. J.* **2022**, *28*, e202200664.
8. Wen, X.J.;Ran, Z.Q.;Zheng, R.X.;Du, D.Y.;Zhao, C.;Li, R.J.;Xu, H.Y.;Zeng, T.;Shu, C.Z. $NiSe_2$@NiO heterostructure with optimized electronic structure as efficient electrocatalyst for lithium-oxygen batteries. *J. Alloy. Compd.* **2022**, *901*, 163703.
9. Wang, Y.;Jiao, Y.Q.;Yan, H.J.;Yang, G.C.;Tian, C.G.;Wu, A.P.;Liu, Y.;Fu, H.G. Vanadium-incorporated CoP_2 with lattice expansion for highly efficient acidic overall water splitting. *Angew. Chem. Int. Ed.* **2022**, *61*, e202116233.
10. Zeng, J.R.;Chen, W.H.;Zhang, G.W.;Yu, L.;Zhong, L.B.;Liu, Y.;Zhao, S.F.;Qiu, Y.J. Heterostructured Ni_3N–NiMoN nanowires as bifunctional electrocatalysts for hydrogen evolution and 5-hydroxymethylfurfural oxidation. *ACS Appl. Nano Mater.* **2022**, *5*, 7321-7330.
11. Li, X.P.;Zheng, L.R.;Liu, S.J.;Ouyang, T.;Ye, S.Y.;Liu, Z.Q. Heterostructures of NiFe LDH hierarchically assembled on MoS_2 nanosheets as high-efficiency electrocatalysts for overall water splitting. *Chin. Chem. Lett.* **2022**, *33*, 4761-4765.
12. Wang, S.G.;Li, J.H.;Fang, H.;Li, B.Y.;Wang, G.M.;Gao, Y.A. 3D core-shell structured NiFe layered double hydroxide with $NiCo_2O_4$ as an efficient

electrocatalysts for oxygen evolution reaction. *J. Phys. Chem. Solids.* **2022**, *166*, 110730.

13. Zhu, S.W.;Wang, J.M.;Li, H.J.;Cai, J.J.;Li, Y.T.;Hu, J.;He, Y.T.;Zhou, Y. NiFe layered double hydroxide nanosheets anchored on cobalt nanocrystal matrixes as electrocatalysts for oxygen evolution. *ACS Appl. Nano Mater.* **2022**, *5*, 13047-13054.
14. Li, J.J.;Qin, Y.N.;Lei, Y.;Li, S.F.;Li, L.;Ouyang, B.;Kan, E.j.;Zhang, W.M. Three-dimensional hierarchical conductive metal–organic frameworks/NiFe layered double hydroxide/carbon nanofibers: an efficient oxygen evolution reaction catalyst for Zn–air batteries. *Inorg. Chem. Front.* **2022**, *9*, 5335-5346.
15. Wen, H.;Yi, Z.Y.;Hu, Z.Y.;Guo, R.;Liu, X.W. Design strategy for low-temperature sulfur etching to achieve high-performance hollow multifunctional electrode material. *J. Mater. Sci. Technol.* **2022**, *119*, 209-218.
16. Li, X.;Patil, K.;Babar, P.;Agarwal, A.;Chen, X.;Myeong Kim, D.;Kim, J.H.;Tae Yoo, Y. Effect of iron concentration and annealing conditions on the catalytic performance of Co–Mn spinel oxides with a unique nanowire–nanosheet coexisting structure for water oxidation. *Energy Fuels* **2022**, *36*, 7806-7815.
17. Liu, Y.X.;Bai, Y.;Han, Y.;Yu, Z.;Zhang, S.M.;Wang, G.H.;Wei, J.H.;Wu, Q.B.;Sun, K.N. Self-supported hierarchical FeCoNi-LTH/$NiCo_2O_4$/CC electrodes with enhanced bifunctional performance for efficient overall water splitting. *ACS Appl. Mater. Interfaces* **2017**, *9*, 36917-36926.
18. Truong, L.;Roy, S.B.;Jerng, S.-K.;Jeon, J.H.;Lee, S.H.;Chun, S.-H. Facile electrodeposition of V-doped CoP on vertical graphene for efficient alkaline water electrolysis. *RSC Adv.* **2020**, *10*, 13016-13020.
19. Shen, J.Y.;Zhang, J.L.;Zhang, G.N.;Li, W.H.;Zheng, M.;Guo, F.Y.;Chen, Q.Q. Interconnected MoS_2/$FeCo_2S_4$ nanosheet array bifunctional electrocatalysts grown on carbon cloth for efficient overall water splitting. *New J. Chem.* **2022**, *46*, 16419-16425.
20. Yang, H.Y.;Dai, G.L.;Chen, Z.L.;Wu, J.;Huang, H.;Liu, Y.;Shao, M.W.;Kang, Z.H. Pseudo-periodically coupling Ni-O lattice with Ce-O lattice in ultrathin heteronanowire arrays for efficient water oxidation. *Small* **2021**, *17*, 2101727.
21. Xu, J.C.;Rong, J.;Zheng, Y.H.;Zhu, Y.;Mao, K.L.;Jing, Z.F.;Zhang, T.;Yang, D.Y.;Qiu, F.X. Construction of sheet-on-sheet hierarchical MoS_2/NiS_2 heterostructures as efficient bifunctional electrocatalysts for overall water splitting. *Electrochim. Acta* **2021**, *385*, 138438.
22. Li, S.S.;Liu, Y.J.;Wu, Y.D.;Du, X.H.;Guan, J.B.;Wang, L.N.;Zhang, M. Tungsten promoted nickel phosphide nanosheets supported on carbon cloth: an efficient and stable bifunctional electrocatalyst for overall water splitting. *Int. J. Hydrog. Energy* **2021**, *46*, 37152-37161.
23. Zhang, X.Y.;Qiu, Y.L.;Li, Q.;Liu, F.G.;Ji, X.Q.;Liu, J.Q. Facile construction of heterostructural $Ni_3(NO_3)_2(OH)_4$/CeO_2 bifunctional catalysts for boosted overall water splitting. *Int. J. Hydrog. Energy* **2022**, *47*, 23221-23229.
24. Wei, X.D.;Chai, Y.D.;Chen, W.F.;Liu, N.;Qiao, S.Y. $Co(OH)_2$-$Ni(OH)_xS_y$ amorphous composite electrocatalysts prepared by electrodeposition method for

efficient oxygen evolution reaction. *Ionics* **2022**, *28*, 3945-3956.
25. Li, S.J.;Xie, W.F.;Song, Y.K.;Shao, M.F. Layered double hydroxide@polydopamine core–shell nanosheet arrays-derived bifunctional electrocatalyst for efficient, flexible, all-solid-state Zinc–air battery. *ACS Sustain. Chem. Eng.* **2020**, *8*, 452-459.
26. Lai, C.L.;Fang, J.Y.;Liu, X.P.;Gong, M.X.;Zhao, T.H.;Shen, T.;Wang, K.L.;Jiang, K.;Wang, D.L. In situ coupling of NiFe nanoparticles with N-doped carbon nanofibers for Zn-air batteries driven water splitting. *Appl. Catal. B Environ.* **2021**, *285*, 119856.
27. Li, S.X.;Zhang, H.;Wu, L.;Zhao, H.W.;Li, L.X.;Sun, C.G.;An, B.G. Vacancy-engineered CeO_2/Co heterostructure anchored on the nitrogen-doped porous carbon nanosheet arrays vertically grown on carbon cloth as an integrated cathode for the oxygen reduction reaction of rechargeable Zn–air battery. *J. Mater. Chem. A* **2022**, *10*, 9858-9868.
28. Yin, M.M.;Miao, H.;Chen, B.;Hu, R.G.;Xia, L.;Zhang, C.F.;Wang, F.;Zhang, H.C.;Yuan, J.L. Self-supported metal sulfide electrode for flexible quasi-solid-state Zinc-air batteries. *J. Alloy. Compd.* **2021**, *878*, 160434.
29. Deeloed, W.;Priamushko, T.;Čížek, J.;Suramitr, S.;Kleitz, F. Defect-engineered hydroxylated mesoporous spinel oxides as bifunctional electrocatalysts for oxygen reduction and evolution reactions. *ACS Appl. Mater. Interfaces* **2022**, *14*, 23307-23321.
30. Liu, Q.;Liu, X.;Xie, Y.;Sun, F.F.;Liang, Z.J.;Wang, L.;Fu, H.G. N-Doped carbon coating enhances the bifunctional oxygen reaction activity of CoFe nanoparticles for a highly stable Zn–air battery. *J. Mater. Chem. A* **2020**, *8*, 21189-21198.
31. Ao, K.L.;Shi, J.H.;Zhang, X.Y.;Daoud, W.A. Tuning oxygen vacancies in spinel nanosheets for binder-free oxygen cathodes with superior catalytic activity in Zinc-air batteries. *J. Power Sources* **2022**, *521*, 230918.
32. Wang, F.L.;Xiao, Z.X.;Liu, X.;Ren, J.W.;Xing, T.;Li, Z.;Li, X.Y.;Chen, Y.L. Strategic design of cellulose nanofibers@zeolitic imidazolate frameworks derived mesoporous carbon-supported nanoscale $CoFe_2O_4$/CoFe hybrid composition as trifunctional electrocatalyst for Zn-air battery and self-powered overall water-splitting. *J. Power Sources* **2022**, *521*, 230925.
33. Wang, M.K.;Liao, T.H.;Zhang, X.L.;Cao, J.L.;Xu, S.Q.;Tang, H.;Wang, Y. Electrospun carbon nanofibers loaded with atomic FeN_x/Fe_2O_3 active sites for efficient oxygen reduction reaction in both acidic and alkaline media. *Adv. Mater. Interfaces* **2022**, *9*, 2101904.
34. Yan, J.H.;Wang, Y.;Zhang, Y.Y.;Xia, S.H.;Yu, J.Y.;Ding, B. Direct magnetic reinforcement of electrocatalytic ORR/OER with electromagnetic induction of magnetic catalysts. *Adv. Mater.* **2021**, *33*, 2007525.
35. Peng, W.;Wang, Y.;Yang, X.X.;Mao, L.C.;Jin, J.H.;Yang, S.L.;Fu, K.;Li, G. Co_9S_8 nanoparticles embedded in multiple doped and electrospun hollow carbon nanofibers as bifunctional oxygen electrocatalysts for rechargeable Zinc-air battery. *Appl. Catal. B Environ.* **2020**, *268*, 118437.
36. zheng, J.H.;Kang, T.J.;Liu, B.;Wang, P.;Li, H.M.;Yang, M. N-doped carbon

nanotubes encapsulated with FeNi nanoparticles derived from defect-rich, molecule-doped 3D g-C_3N_4 as an efficient bifunctional electrocatalyst for rechargeable Zinc–air batteries. *J. Mater. Chem. A* **2022**, *10*, 9911-9921.

37. Li, Y.;Zhou, W.H.;Zheng, L.C.;Liu, J.;Tang, R.R.;Shi, K.J.;Zhang, Y.Y. Hollow porous nitrogen-doped carbon formed by Fe-modified bimetallic organic framework for rechargeable liquid/solid Zn-air batteries. *J. Alloy. Compd.* **2021**, *886*, 161227.
38. Xie, Y.H.;Feng, C.;Guo, Y.;Hassan, A.;Li, S.;Zhang, Y.;Wang, J.D. Dimethylimidazole and dicyandiamide assisted synthesized rich-defect and highly dispersed CuCo-Nx anchored hollow graphite carbon nanocages as efficient trifunctional electrocatalyst in the same electrolyte. *J. Power Sources* **2022**, *517*, 230721.
39. Jin, Q.Y.;Ren, B.W.;Chen, J.P.;Cui, H.;Wang, C.X. A facile method to conduct 3D self-supporting Co-FeCo/N-doped graphene-like carbon bifunctional electrocatalysts for flexible solid-state zinc air battery. *Appl. Catal. B Environ.* **2019**, *256*, 117887.
40. Jin, Q.Y.;Ren, B.W.;Cui, H.;Wang, C.X. Nitrogen and cobalt co-doped carbon nanotube films as binder-free trifunctional electrode for flexible Zinc-air battery and self-powered overall water splitting. *Appl. Catal. B Environ.* **2021**, *283*, 119643.
41. Li, W.J.;Wang, F.;Zhang, Z.G.;Min, S.X. Graphitic carbon layer-encapsulated Co nanoparticles embedded on porous carbonized wood as a self-supported chainmail oxygen electrode for rechargeable Zn-air batteries. *Appl. Catal. B Environ.* **2022**, *317*, 121758.
